# A transcriptome-based phylogenetic study of hard ticks (Ixodidae)

**DOI:** 10.1038/s41598-019-49641-9

**Published:** 2019-09-09

**Authors:** N. Pierre Charrier, Axelle Hermouet, Caroline Hervet, Albert Agoulon, Stephen C. Barker, Dieter Heylen, Céline Toty, Karen D. McCoy, Olivier Plantard, Claude Rispe

**Affiliations:** 1 BIOEPAR, INRA, Oniris, Nantes, France; 2 Department of Parasitology, School of Chemistry & Molecular Biosciences, The University of Queensland, Brisbane, Qld Australia; 3 Evolutionary Ecology Group, Department of Biology, University of Antwerp, Wilrijk, Belgium; 4 Interuniversity Institute for Biostatistics and statistical Bioinformatics, Hasselt University, Diepenbeek, Belgium; 5 Department of Ecology and Evolutionary Biology, Princeton University, Princeton, NJ USA; 6 Laboratoire MIVEGEC (Maladies Infectieuses et Vecteurs: Ecologie, Génétique, Evolution & Contrôle), Université de Montpellier, Centre National de la Recherche Scientifique (UMR5290), Institut de Recherche pour le Développement (UR224), Montpellier, France

**Keywords:** Phylogenetics, Evolutionary biology

## Abstract

Hard ticks are widely distributed across temperate regions, show strong variation in host associations, and are potential vectors of a diversity of medically important zoonoses, such as Lyme disease. To address unresolved issues with respect to the evolutionary relationships among certain species or genera, we produced novel RNA-Seq data sets for nine different *Ixodes* species. We combined this new data with 18 data sets obtained from public databases, both for *Ixodes* and non-*Ixodes* hard tick species, using soft ticks as an outgroup. We assembled transcriptomes (for 27 species in total), predicted coding sequences and identified single copy orthologues (SCO). Using Maximum-likelihood and Bayesian frameworks, we reconstructed a hard tick phylogeny for the nuclear genome. We also obtained a mitochondrial DNA-based phylogeny using published genome sequences and mitochondrial sequences derived from the new transcriptomes. Our results confirm previous studies showing that the *Ixodes* genus is monophyletic and clarify the relationships among *Ixodes* sub-genera. This work provides a baseline for studying the evolutionary history of ticks: we indeed found an unexpected acceleration of substitutions for mitochondrial sequences of Prostriata, and for nuclear and mitochondrial genes of two species of *Rhipicephalus*, which we relate with patterns of genome architecture and changes of life-cycle, respectively.

## Introduction

Ticks are blood-feeding arthropods which parasitize terrestrial vertebrates, including mammals, birds, lizards and snakes. They are a concern in human and animal health notably for their potential to transmit infectious agents (including bacteria, viruses, protozoa and nematodes). Understanding phylogenetic relationships among tick species is an important step to better apprehend biological differences among genera or species, including traits related to host specificity and to pathogen transmission. Extant ticks (Ixodida) are divided into two principal families, the soft ticks (Argasidae, with ~200 species) and the hard ticks (Ixodidae, with ~700 species) whith an additional monospecific family (Nutalliellidae)^1,2^ that appears to be a sister-group to the ensemble formed by hard ticks and soft ticks, and shares traits with these two groups. Hard ticks can be further divided into two groups based on morphological traits, the Metastriata and Prostriata, with respectively ~450 species and ~250 species^3^. While there is a general consensus on the classification of hard tick genera (largely based on morphological traits) and significant advances have been made using molecular approaches^4,5^, some unresolved issues with respect to the placement of certain species and the evolutionary relationships among genera remain.

The aim of the present study is to examine the phylogenetic relationships among ticks, with a special focus on the *Ixodes* genus, motivated by the fact that this genus comprises several species with strong impacts on animal and human health (for example vectors of the Lyme disease agent, *Borrelia burgdorferi* s.l. are all *Ixodes* species. Debate surrounded the initial phylogenies of the Ixodidae produced using morphology^4,5^, but there is now a broad consensus about the deep-level branches in the tick evolutionary tree and about the contour of most genera^6,7^. Recent studies^8,9^ have used a combination of markers (including nuclear rRNA sequences and complete mitochondrial genome sequences (mitogenomes) to build robust phylogenetic trees for ticks. But these studies focused either on soft ticks or on Metastriata, a subgroup of hard ticks. By comparison, less complete and in-depth molecular phylogenetic studies have been produced for the genus *Ixodes*. The 244 described species of the *Ixodes* genus are organized into numerous sub-genera, between 14 and 18 depending on authors^10,11^. While recognized as coherent from a systematic point of view^12^, some of these sub-genera are still debated and several species have not yet been assigned to a sub-genus (e.g. *I*. *acuminatus*, *I*. *ventalloi*)^10,11^. In a recent study^13^, it was shown for example that the Eschatocephalus subgenus (species associated with bats) clusters within the subgenus Pholeoixodes (which was therefore paraphyletic). Likewise, a question that has elicited some debate is the relationship between the Australasian *Ixodes* lineage and the other species as several studies have questioned the monophyly of the *Ixodes* genus^14–16^. Recent results based on complete mitogenomes^8,9^ support a monophyletic *Ixodes* genus, but highlight an early divergence between the Australasian *Ixodes* lineage and other species. In the present study, we attempt to resolve the phylogeny of hard ticks (in particular within the *Ixodes* genus) using a more powerful transcriptomic approach and comparing trees generated with nuclear and mitochondrial genes. An increasingly common approach in molecular systematics consists in sequencing transcriptomes with high throughput methods (RNA-Seq) which allows to reconstruct large gene collections for each species, providing data for robust phylogenetic construction^17,18^ in addition to precious genomic resources for each species. This approach has been used to construct phylogenies in different groups, e.g. Bivalva^19^, Arachnida^20,21^, Myriapoda^22^ or Hymenoptera^23^ and has even been applied to resolve the phylogeny of closely related taxa^24^. There has been a rapid accumulation of transcriptome studies for ticks in recent years^25^. However, to our knowledge, analyses of these transcriptomes have essentially been performed on a per species basis, with the objective to explore mechanisms implicated in the feeding process, blood digestion or interactions with pathogens. A recent study^26^ was the first to propose a transcriptome-based phylogeny of ticks, but included only a limited number of species and focused on soft ticks. Overall, most RNA-Seq data concern Metastriata and soft ticks, whereas few species of Prostriata (*I*. *scapularis*, *I*. *ricinus*, *I*. *persulcatus* and *I*. *holocyclus*) have been sequenced for their transcriptomes. To obtain a broader and more robust phylogenetic scenario for tick evolution, there was thus a need to produce more transcriptomes for *Ixodes* species. In order to clarify the phylogenetic relationship among hard tick species, we produced new RNA-Seq data for nine species of the *Ixodes* genus -this list includes *I*. *holocyclus* for which another research group recently and independently sequenced a transcriptome^27^. We also used previously published RNA-Seq data sets for other ticks, including Metastriata and soft tick species, for a total of 27 different species in our phylogenetic analysis. For species with previously published transcriptomes, we reassembled the raw data and used a prediction pipeline with the same homogeneous method for all species. We thereby obtained thousands of coding genes for each species and hundreds of orthologous genes among species. Using this RNA-Seq data, we then produced a robust phylogenetic framework for the hard ticks, comparing the transcriptome-based nuclear phylogeny with that of mitogenomes. In doing so, we significantly enriched molecular and phylogenetic data for the *Ixodes* genus and were able to resolve both the general issues outlined above and some specific issues raised during the analyses.

## Results

### Sequencing statistics

In the present study, we produced new transcriptomes for nine different species of *Ixodes* (Table 1). For *I*. *acuminatus*, *I*. *arboricola*, *I*. *canisuga*, *I*. *frontalis*, *I*. *hexagonus*, *I*. *holocyclus*, *I*. *ventalloi*, and *I*. *vespertilionis*, we obtained between 29.2 and 43.9 M paired-end (PE) reads (Table 2). We also generated 225.8 M PE reads for *I*. *uriae* in an independent sequencing run. For the published data sets from the 18 other tick species, the number of reads ranged between 6.8 M (*Rhipicephalus appendiculatus*) and 204.8 M (*Ornithodoros erraticus*). Read length varied among projects (between 76 and 300) which also contributed to the variation of the total sequence output among species.

**Table 1:** Taxonomic and sampling details for nine tick species of the genus *Ixodes*, used for an RNA-seq based phylogenetic study.

Name	Sub-genus	Host	Collection site	Composition
*I*. *acuminatus*	Ixodes Latreille, 1795	Wood mouse (*Apodemus sylvaticus*)	Pleine-Fougères, France	10FU, 10M, 49NU, 45NE
*I*. *arboricola*	Pholeoixodes Shulze, 1942	Great tits (*Parus major*)	Antwerp, Belgium	6FU, 3FE, 5M, 17NE
*I*. *canisuga*	Pholeoixodes Shulze, 1942	Red fox (*Vulpes vulpes*)* - Badger (*Meles meles*)	Grotte de Chanzelle, France - Nantes, France	6FU, 3FE, 5M, 7NU
*I*. *frontalis*	Trichotoixodes Reznik, 1961	Black bird (*Turdus merula*)	Nantes, France	5FE, 9NE
*I*. *hexagonus*	Pholeoixodes Shulze, 1942	Hedgehog (*Erinaceus europeus*)	Nantes, France	2FU, 1FE, 15NU, 5NE
*I*. *holocyclus*	Sternalixodes Schulze, 1935	Questing ticks	Queensland, Australia	10FU
*I*. *uriae*	Ceratixodes Neumann, 1902	Common murres (*Uria aalge*)	Hornoya, Norway	FU, M, NU, NE
*I*. *ventalloi*	ungrouped	European rabbit (*Oryctolagus cuniculus*)	Ile d'Houat, France	4FU, 3FE
*I*. *vespertilionis*	Eschatocephalus von Frauenfeld, 1853	Lesser horseshoe bat (*Rhinolophus hipposideros*)	Grotte de Chanzelle, France	4FU

The subgenus grouping is based on either Clifford *et al*.^10^ and/or Camicas *et al*.^11^. The subgenus assignation of *I*. *fontalis* is that of Camicas, which differs from Clifford’s. Host: an asterisk indicates that the host was inferred, ticks having been collected in caves frequented by a determined mammalian species. Collection site corresponds to the name of the locality or region followed by the name of the country. Composition lists the life stages and physiological condition of ticks used to generate the library: FU-female, unengorged; FE-female, engorged; M-male; NU-nymph, unengorged; NE-nymph, engorged, preceded by the number of individuals used for RNA extraction. For *I*. *uriae*, a library was obtained independently for each life stage and physiological condition.

**Table 2:** Sequencing and assembly statistics for the transcriptomes of 27 species used in this study.

Species	Depth (M reads)	# peptides	# BUSCO	percent Busco
*A*. *americanum*	113.09	27189	922	34.47
*A*. *maculatum*	48.29	11125	568	21.23
*A*. *sculptum*	95.75	22578	982	36.71
*D*. *andersoni*	57.45	27262	1763	65.91
*D*. *variabilis*	71.72	27066	1806	67.51
*Ha*. *flava*	79.91	57981	1890	70.65
*Hy*. *excavatum*	130.73	14505	661	24.71
*I*. acuminatus*	29.24	20250	764	28.56
*I*. arboricola*	41.82	22179	1735	64.86
*I*. canisuga*	38.97	15283	881	32.93
*I*. frontalis*	42.03	18187	1069	39.96
*I*. hexagonus*	43.91	3215	130	4.86
*I*. holocyclus*	37.26	15520	1321	49.38
*I*. *persulcatus*	105.95	41620	1891	70.69
*I*. *ricinus*	162.87	71841	1936	72.37
*I*. *scapularis*	357.46	63018	1973	73.76
*I*. uriae*	225.79	49056	2074	77.53
*I*. ventalloi*	38.59	16563	997	37.27
*I*. vespertilionis*	33.63	21090	1133	42.36
*O*. *erraticus*	204.79	43106	2056	76.86
*O*. *moubata*	110.61	12839	1845	68.97
*O*. *rostratus*	52.18	25454	1830	68.41
*R*. *annulatus*	31.00	28256	1418	53.01
*R*. *appendiculatus*	12.18	35728	1689	63.14
*R*. *microplus*	62.73	23471	926	34.62
*R*. *pulchellus*	55.29	46076	1219	45.57
*R*. *zambeziensis*	42.93	61008	1759	65.76
**Mean**	86.16	30425	1379	51.56
**SD**	76.20	17843	537	20.09

Columns: species names (an asterisk indicates new transcriptomes generated for this study), depth (number of million trimmed and cleaned reads), number of predicted peptides, number of complete BUSCO genes (using the Arthropod BUSCO v1 data set), percentage of completeness.

### Assembly and gene prediction

Raw assemblies comprised between 23,992 (*I*. *hexagonus*) and 517,072 contigs (*I*. *scapularis*), with an average of 30,425 contigs per species (Table 2). We predicted between 4613 peptides for *I*. *hexagonus* and 289,763 peptides for *I*. *scapularis*, with an average of 96,268 predicted peptides per species. A compression step reduced the number of predicted peptides by 61.3% on average. Completeness of these reduced assemblies was assessed with the BUSCO approach. This metric ranged between 4.86% for *I*. *hexagonus* and 77.53% for *I*. *uriae* with an average completeness of 51.56% (Supplementary Fig. S1). We stress that completeness metrics vary with the version of the BUSCO lineages data sets: we indeed have used the BUSCO Arthropod v1 data set (with 2675 genes) as it was more complete (and, we assumed, as it had more power to compare assemblies); with the more restrictive Arthropod v2 data set (1066 genes), completeness reached 95.5% for *I*. *ricinus* instead of 72%. We tested if the variability of completeness was related to the used of cleaned read pairs, log10 transformed (F_(1,25)_ = 4.23, *p* = 0.05, a relationship just at the threshold of significance). This relationship became statistically significant when read length was included as an explanatory variable in the model (F_(2,24)_ = 4.21, *p* = 0.027). This multiple regression explained almost 20% of the variability in completeness (adjusted R^2^ = 0.198) (Supplementary Fig. S2). The number of complete BUSCO genes tended to plateau above 50 M reads, indicating a saturation of the sequence information in larger data sets. For smaller data sets, completeness varied strongly among projects (possibly related to the diversity of conditions used, but also to variation in RNA quality). For example, we assume that a quality issue explains the very low completeness of the *I*. *hexagonus* data set.

### Identification of orthologous genes

**Figure 1:**
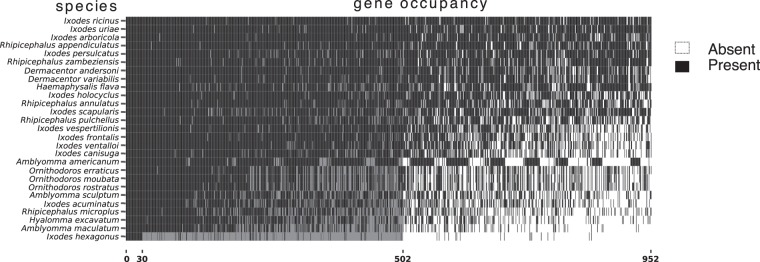
Single Copy Orthologues (SCO) occupancy matrix, generated after clustering with Silix. Each row represents a species (from top to bottom, species with a decreasing % of occupancy), while each column represents a SCO (presence of the gene is indicated by a black-filled cell). Different levels of shading indicate occupancy level: left (columns 1 to 30), SCO present in 100% of the species, center (columns 31 to 502), SCO present in between 75% and 100% of the species, right (columns 503 to 952), SCO present in between 50% and 75% of the species.

The optimal clustering parameters with SiLix were an identity of 75% and an overlap of 75% (SCO100, Supplementary Fig. S3). Other parameters (partial overlap and minimum length) did not have an impact on the number of SCO100. These parameters were set to a partial overlap of at least 200 amino acid positions and an overlap superior to 90%. For these parameters, SiLiX predicted 30 SCO100 (single copy genes shared by all 27 species), 502 SCO75 (present at least in 75% of the species), and 952 SCO50 (present at least in 50% of the species) - Fig. 1.

### Alignment

Two genes (out of 502) from the SCO75 matrix showed signs of saturation and were discarded. The total number of nucleotide sites obtained for the three supermatrices (SCO100, SCO75, and SCO50) were respectively 20,094 (0% missing data - 11,417 constant sites), 397,632 (11.7% missing data - 221,141 constant sites), and 824,406 (25.5% missing data - 460,607 constant sites). The percentage of missing data per species for the SCO50 supermatrix ranged between 4.42% for *I*. *uriae* and 92.18% for *I*. *hexagonus*.

### Phylogeny based on nuclear sequences

**Figure 2:**
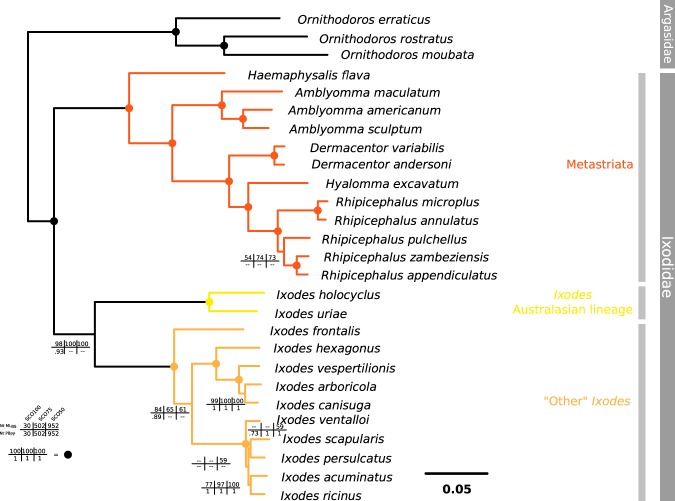
Phylogenetic tree based on nuclear genes, using the SCO50 supermatrix, mid-point rooted. Nodes with dots represent maximum support values (100% in all methods), otherwise bootstrap value and posterior probability were represented. These values are indicated for each of the SCO matrixes (occupancy level of 100%, 75%, or 50%): bootstrap values with the ML method are indicated on the top line, and posterior probabilities for the Bayesian approach on the bottom line.

**Figure 3:**
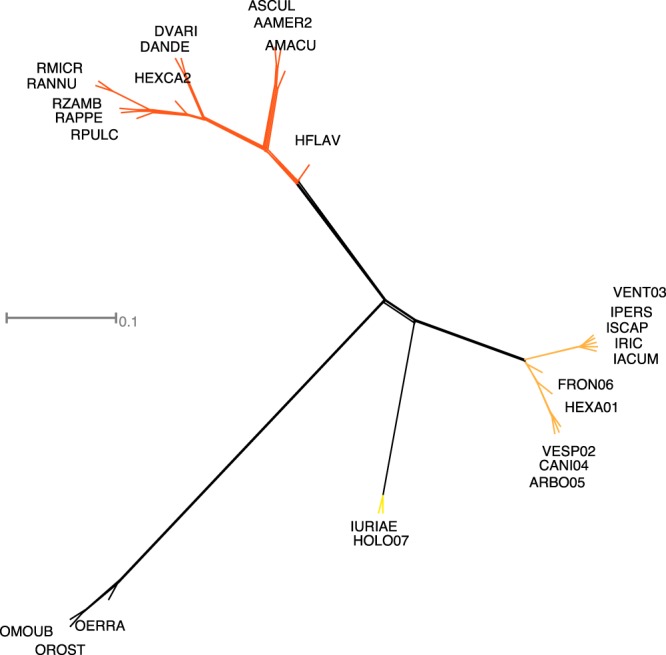
Supernetwork based on genes from the SCO75 matrix (occupancy level of 75% among 27 tick species). This supernetwork was built from nucleotide alignments, including all codon positions. Species names abbreviated for readability (key of complete name in Supplementary Table S3).

Convergence for the Bayesian analysis was obtained for each of the three supermatrices (with effective sizes >100 and MaxDiff <0.10 for most parameters, see Supplementary Table S3). For both ML and Bayesian methods, we found the following topology (Fig. 2): the Metastriata formed a monophyletic clade (100% bootstrap or posterior probability support, in all three supermatrices). There was also strong support for the monophyly of the Prostriata (i.e. the *Ixodes* genus) both with the ML method (all three matrices) and with the Bayesian approach (with the SCO100 matrix only). Unexpectedly, the Bayesian approach gave different topologies for SCO75 and SCO50 matrices (based on more sites overall, but with some incompleteness), in which *I*. *uriae* and *I*. *holocyclus* species formed a sister group of the Metastriata (but with low support). With the Bayesian approach, the branch lengths separating the three lineages (Metastriata, Australasian *Ixodes* and the non-Australasian *Ixodes*) were very short with 0.019 and 0.010 respectively, for SCO75 and SCO50. With all methods, there was a clear separation within the *Ixodes* genus between the Australasian lineage on one side (represented by *I*. *holocyclus* and *I*. *uriae*) and a second clade comprising all other species. The distance between Australasian species and other *Ixodes* species (0.270) was only slightly shorter than distances between the Metastriata and any *Ixodes* species (Table 3). Within the Metastriata, *Haemaphysalis flava* was found to be the sister group of all other Metastriata (supported in all analyses). We also found that *Hyalomma excavatum* was embedded in the *Rhipicephalinae* subfamily (represented here by two genera, *Dermacentor* and *Rhipicephalus*). While the different *Rhipicephalus* species formed a robust clade, the position of *R*. *pulchellus* was not robust among methods, nor among data sets. Among the non-Australasian *Ixodes* lineage, the bird-associated species *I*. *frontalis* appeared as a sister group to all other species, which were themselves divided into two well supported clades. The first one comprised four *Ixodes* species (*I*. *hexagonus*, *I*. *vespertilionis*, *I*. *arboricola*, *I*. *canisuga*), while the second comprised the five remaining species (*I*. *ventalloi*, *I*. *scapularis*, *I*. *persulcatus*, *I*. *acuminatus* and *I*. *ricinus):* these species were particularly close to each other and we could only determine a robust grouping within this clade between *I*. *acuminatus* and *I*. *ricinus*. The supernetwork from the SCO75 matrix based on the nucleotide alignments including all codon positions is represented in Fig. 3. The different supernetworks, corresponding to the nine different combinations of sites or matrices, showed the same topology (data not shown), which also reflected the results of the ML and Bayesian approaches.

**Table 3:** Genetic distance among lineages (patristic distance), for nuclear genes, defined as the sum of branch lengths between taxa.

Lineage	*Amblyomma*	*Dermacentor*	*Haemaphysalis*	*Hyalomma*	*“other”-Ixodes*	*Aust.-Ixodes*	*Ornithodoros*	*Rhipicephalus*
*Amblyomma*	.	0.174	0.196	0.193	0.348	0.348	0.426	0.200
*Dermacentor*	0.005	.	0.204	0.109	0.356	0.356	0.434	0.116
*Haemaphysalis*	0.006	>0.001	.	0.223	0.307	0.307	0.386	0.230
*Hyalomma*	0.006	>0.001	na	.	0.375	0.375	0.453	0.103
*“other”-Ixodes*	0.007	0.006	0.006	0.006	.	0.270	0.415	0.382
*Aust.-Ixodes*	0.006	0.003	0.004	0.004	0.006	.	0.415	0.382
*Ornithodoros*	0.018	0.018	0.020	0.020	0.017	0.018	.	0.460
*Rhipicephalus*	0.009	0.008	0.008	0.008	0.009	0.008	0.018	.

The upper-right corner gives the mean distance between lineages, while the bottom-left corner gives standard deviations (“na” means “not available”). Distances were computed from the ML consensus tree of the SCO50 data set.

### Phylogeny based on mitochondrial protein sequences

After extraction of potential contaminants, we could unambiguously obtain a complete or near complete sequence for all genes of each species (among the nine targeted). In the case of *I*. *frontalis*, we were confronted with the presence of two divergent gene sequences. This observation is line with a recent finding^28^ of two divergent lineages co-occuring in Hungary. For *cox1*, the full-length alignment of the two variants revealed an anomaly; it combined regions of moderate divergence and regions of complete identity, suggesting chimerism in the contigs produced by Trinity. To solve this problem, we extracted reads that matched the *cox1* sequence and reassembled them with CAP3^29^, using stringent identity criteria to separate each variant. We obtained two full-length sequences, characterized by moderate divergence over the full gene sequence (thereby solving the initial chimerism problem). A phylogenetic analysis including several closely related species in the *Ixodes* genus, with the same methods as described above confirmed the deep divergence between these two mitochondrial (mt) lineages (Supplementary Fig. S4) far exceeding that between the most divergent mitogenomes of *I*. *ricinus* (IR3_TN and IR13_TN)^30^, and approaching the divergence between two species (*I*. *ricinus* versus *I*. *acuminatu*s). We did not reproduce this analysis for the eight remaining mt genes of *I*. *frontalis*, since it would have been impossible to phase the different variants. We thus chose to randomly select one of the two variants obtained for each gene, keeping in mind that they might represent a mixture of two divergent mt lineages.

**Figure 4:**
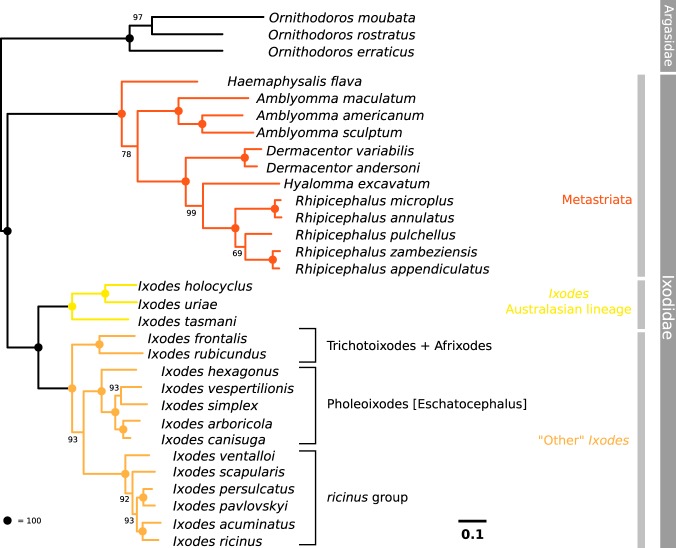
Phylogenetic tree based on nine concatenated mitochondrial genes (mtAA9 data set)). Mitochondrial sequences were either derived from published mitogenomes, or if not available, from *de novo* transcriptome assembly (extracting contigs and translating ORFs). The tree was obtained with an ML method, with model optimization for each gene, as described in the text, with bootstrap support at the nodes.

For the different mt proteins (gene name between parentheses), the best-fit model according to BIC was: mtMet + F + I + G4 (*atp6*, *nad2*), mtART + I + G4 (*cox1*), mtMet + I + G4 (*cox2*, *cytb*), mtART + G4 (*cox3*), mtInv + I + G4 (*nad1*, *nad4*, *nad5*). Phylogenetic analysis of the mtAA9 data set essentially replicated that of the analysis based on nuclear genes (Fig. 4). The only difference concerned the fact that *I*. *scapularis* was found to be a sister species of *I*. *persulcatus* with the nuclear genes (with low support), but not with mitochondrial data set. In the mt phylogenetic study, all branches showed strong bootstrap support, with the single exception of the position of *R*. *pulchellus* within the *Rhipicephalus* genus.

Our new data led us to re-examine the unexpected intraspecific variability found in a previous study of *I*. *ricinus* mitogenomes^30^. We compared several mitogenomes of *I*. *ricinus* published in this study, along with the first published mitogenome for the specie*s* (JN248424)^31^ and other *Ixodes* species based on the new transcriptome sequences. Our reanalysis of the *cytb* gene suggests that JN248424 represented a chimeric assembly between two species, *I*. *ricinus* and *I*. *acuminatus* (Supplementary Fig. S5).

Interestingly, the mtAA9 tree indicated longer branches in one subgroup (Metastriata) compared to the other (Prostriata). We therefore compared evolutionary rates among these groups, using the soft tick species (*Ornithodoros rostratus*) as an outgroup. We found (Supplementary Table S4) that there was a systematic and significant excess of substitutions in the Metastriata (there was on average 1.58 times more unique substitutions in Metastriata than in Prostriata). This suggests a shift in molecular rates of evolution between the two groups, with an acceleration of evolutionary rates in the Metastriata or a deceleration in the Prostriata with respect to Argasid ticks. We extended the same approach to nuclear genes, selecting the SCO100 data set (n = 30 single copy orthologues present in the 27 species) to check if this obervation also applied to the nuclear coding genes. This time, most of the comparisons were not significant, with the exception of comparisons including *Rhipicephalus microplus*, suggesting an acceleration of evolutionary rates restricted to one or a few species of the genus *Rhipicephalus*, but not in Metastriata as a whole. Finally, we compared evolutionary rates of the two species of *Rhipicephalus* that form the Boophilus subgenus with other species of *Rhipicephalus*: we found that mitochondrial genes and nuclear genes showed significantly more substitutions in Boophilus species (Supplementary Table S4) - by factors ~1.5 and 1.8 for mitochondrial and nuclear genes, respectively.

## Discussion

Our study aimed at obtaining a robust phylogeny of the hard ticks, with a focus on the Prostriata (the *Ixodes* genus). We sequenced new transcriptomes for nine species of *Ixodes* (eight of which represent the first published data sets), and combined this data with public RNA-Seq data sets, principally for the Metastriata. For the Metastriata, our transcriptome-based phylogeny recapitulated the results of mitogenome based studies^6–8,32^; we will therefore not comment further on the grouping of species or genera for this group. We stress, however, the interest of using two different sets of markers (nuclear versus mitochondrial) and comparing their results. Over relatively long evolutionary time periods, it is not surprising to find the same topology with both types of markers. However, if we consider groups that have diverged more recently, and that comprise species which show traces of recent hybridization (between them or with other species not represented in the data set), the two signals could differ (see for example the case of *I*. *frontalis* in the results). Our broad conclusion is that transcriptomes, even when sequenced at a relatively low coverage (i.e. ~30 M reads), can yield enough information for phylogenetic reconstruction. We also showed that assembled contigs contained most of the coding mitogenome (full-length mitochondrial CDSs could be found for nearly all species included in our study, as also shown in a recent study^9^), which was also used to make phylogenetic inferences.

The monophyletic character of *Ixodes* has been under debate^32^. It has been noted that both the Australasian *Ixodes* lineage and Metastriata have a duplicated control region (CR)^33,34^. But the genomic localization of the duplicate copies is not the same in Australasian *Ixodes* and in the Metastriata^33^, suggesting independent duplication events rather than common ancestry. Our transcriptome-based phylogeny, using several hundred shared nuclear genes, supports *Ixodes* as a monophyletic group overall. All supernetworks were essentially identical despite they were built with different types of alignments. The Bayesian approach led to the same result as the ML approach only for the SCO100 matrix. With the SCO75 and SCO50 supermatrices, the Australasian *Ixodes* lineage and the Metastriata formed a sister group. However this result is associated with very short internal branches separating the Metastriata, Australasian and non-Australasian-*Ixodes* lineages. To explain this discrepancy, we propose that the SCO50 matrix included less conserved sequences, with higher substitution rates compared to more complete matrices. Indeed, other studies have shown that analyses including subsets of genes with comparatively higher rates of substitution led to inconsistent phylogenetic reconstructions, due to problems of long branch attraction^20,35^. Alternatively, the discrepancies between topologies could be a direct product of the inclusion of missing data, which can lead to instability during phylogenetic reconstruction, as different methods (ML and Baysian) may deal differently with missing sequences, as shown in previous examples^36,37^. Here, given the strong support found with the ML approach (for all matrices) and for the Bayesian approach with the SCO100 matrix, and considering the additional support from the mitochondrial information^7,32^, we may conclude that *Ixodes* is monophyletic, with two deeply divergent subgroups (Australasian species versus the other species). Within the ‘other *Ixodes sp*.’ group, we could define (i) a basal group comprising *I*. *frontalis* (in the Trichotoixodes subgenus) and *I*. *rubicundus* (in the Afrixodes subgenus), (ii) a group comprising several species that belong to the Pholeoixodes subgenus or to the Eschatocephalus subgenus and (iii) a group of closely related species comprising *I*. *ricinus*. For the Pholeoixodes + Eschatocephalus group, our study supports the findings from a phylogenetic study^13^ which showed that the subgenus Eschatocephalus (a group of species associated with bats) were imbedded in another subgenus, Pholeoixodes,which is therefore paraphyletic. This result illustrates the rapid emergence of new host associations. The expression ‘ricinus complex’ was initially used to describe three species (*I*. *ricinus*, *I*. *persulcatus* and *I*. *scapularis*), sharing similar morphologies, life-styles and the ability to transmit the Lyme disease spirochaetes^38^. Even though closely related species are unable to transmit this pathogen^38,39^, the term ‘ricinus group’ is still used^39–41^ to describe this group of closely related species. Our analysis (considering both the nuclear and mitochondrial phylogenies) confirms the extreme relatedness of six species (*I*. *ricinus*, *I*. *acuminatus*, *I*. *persulcatus*, *I*. *pavlovskyi*, *I*. *scapularis* and *I*. *ventalloi*) that should be considered within the ‘ricinus group’ and questions the use of criteria related to vector competence for defining evolutionary relationships. Although not included in the present study, we would also include *I*. *pacificus* in the ‘ricinus group’ as it is known to be closely related to the others^42^. The ensemble *I*. *ricinus* + *I*. *persulcatus* + *I*. *pacificus* + *I*. *scapularis* likely represents a case of vicariance, since these four species cover largely non-overlapping regions of the Palearctic and Neoarctic^12^ and have similar ecologies (e.g. exophylic life-cycles, polyphagy and high abundance in their ecosystem). Based on their phylogenetic position, we would also include two novel western European species in the ‘ricinus group’: *I*. *acuminatus* and *I*. *ventalloi*. Both species had remained unassigned to the subgenus level in previous studies^11^. Although differing in some ecological traits, *I*. *acuminatus* was indeed found to be the sister species of *I*. *ricinus*. Interestingly, this species was predicted to have a high probability of transmitting infections from animal hosts to humans in a recent modelling study^43^. *I*. *acuminatus* and *I*. *ventalloi* are strongly, but not exclusively, associated with small rodents and rabbits, respectively. Given the short genetic distances between species, the ‘ricinus group’ as defined here, illustrates a case of rapid radiation associated, at least in part, with changes in patterns of host specialization.

One potential problem associated with RNA-Seq-based approaches is the incompleteness of transcriptomic data sets: inevitably, the number of shared single copy orthologues is ever decreasing with the number of species (each gene has a risk of not being reconstructed in a given species, or to be incorrectly identified as duplicated due to the redundancy between alternative transcripts). Among the new data sets produced for this study, one of them (*I*. *hexagonus*) yielded a particularly low number of transcripts and of conserved genes (genes used in the SCO matrixes), possibly due to a lower quality RNA (this was suggested by an anomaly in the distribution in percentages of GC in the reads, either suggesting contamination or a high fraction of rRNA). However, the phylogenetic position of this species was very robust and coherent with a recent study of the *Pholeoixodes* subgenus^13^. Another relatively incomplete transcriptome was that of *Hy*. *excavatum* possibly because the sample was restricted to a single tissue, salivary glands^44^. Again, despite this limitation, our methods resulted in the robust placement of this species with respect to the *Rhipicephalinae*. In a study that explored the problem of incomplete supermatrices^45^, the authors concluded that obtaining even a modest number of genes (in the order of 50) in all, or nearly all, species and selecting an adequate model of substitution greatly help avoiding artefacts in phylogenetic reconstruction. We obtained a relatively small SCO100 matrix (with only 30 genes), which was largely due to the two most incomplete gene collections (especially *I*. *hexagonus*), but the number of shared genes rapidly increased when we relaxed the criteria of presence (reaching a total of 502 SCO genes present in 75% of the species). This suggests that the level of completeness was sufficient to allow a robust reconstruction. Interestingly, supernetwork topologies were identical overall regardless of the nature of alignments (amino acid versus DNA, DNA including all codon positions or only positions 1 and 2), but the level of reticulation was higher for amino acids alignments than for nucleotides alignments including all codon positions. This would suggest that the signal contained in the DNA sequences (with more substitutions) enables a finer-scale resolution.

Our gene reconstruction process aimed at obtaining a consensus for each gene in each species, neglecting intraspecific polymorphism. However, for *I*. *frontalis* our mitochondrial DNA analysis revealed a deep divergence between two mitochondrial lineages. This was unexpected given that the sample was composed of a small number of individuals, sampled on a single bird. Two scenarios could explain this situation i) the existence of cryptic species that coexist in sympatry ii) a case of introgression, due to a past hybridization event with a yet undetermined species. We note that the reconstruction of conserved nuclear genes (BUSCO approach) did not lead to any signal of high genetic variability. In fact, the number of BUSCO genes predicted as duplicated was very low in *I*. *frontalis*. Our conclusion is that nuclear genes do not support cryptic species and the mitochondrial data represent evidence of past introgression. Our mitochondrial DNA analysis also demonstrated that one mitogenome published for *I*. *ricinus* resulted from a chimeric assembly of *I*. *ricinus* and *I*. *acuminatus* material, as two regions of the same gene sequence (*cytb*) perfectly matched respectively *I*. *ricinus* or *I*. *acuminatus* (Supplementary Fig. S5). Whether this was caused by misidentification or by contamination of samples after extraction cannot be determined here.

An unexpected finding of our study was the accelerated substitution rate for mt genes in the Metastriata relative to the Prostriata (Table S4). In contrast with mitochondrial genes, there was no significant difference between the two groups for nuclear genes, suggesting that it is not a demographic factor (i.e. generation time, population size) that explains a genome-wide difference in evolutionary rates, as this would affect both mitochondrial and nuclear genes. It has been shown that soft ticks (or Argasidae, the outgroup in our study) and the Prostriata share the same mitochondrial gene order, which is the ancestral order of arthropods^34^. The Metastriata, however, are characterized by a distinct gene order^34,46^. The increased substitution rate in Metastriate ticks could thus be related to the specific arrangement of their mitochondrial genome. More studies, including molecular calibration and estimation of evolutionary rates would help resolve this issue and would help uncover the molecular mechanism of this change. Besides, for both mitochondrial and nuclear genes, we did observe an acceleration of substitution rates in two Metastriata sister species, *R*. *microplus* and *R*. *annulatus*, of the subgenus Boophilus. These species have a distinctive life-cycle trait, completing their entire life-cycle on a single vertebrate host. Such 1-host tick species may have much shorter generation times than other ticks, which would explain these higher evolutionary rates. Our observation is in line with previous studies on *R*. *microplus* demonstrating rapid changes in this species^47,48^.

Altogether, our study shows that RNA-Seq is a promising tool to resolve the phylogenetic relationships among tick groups in a cost-effective way, with both nuclear and mitochondrial genes. For the 27 species considered here with nuclear genes (31 species with mt genes), and with novel data sets produced for several *Ixodes* species, this study has enabled us to confirm or clarify relationships among the main groups of hard ticks, and among the different subgenera of *Ixodes*. The comparison of nuclear and mitochondrial data also revealed unexpected patterns, notably differences in molecular clocks among species and groups. More generally, this work represents a baseline for further studies exploring the impact of tick life-style on gene evolution, providing solid data to test the relationship between life-history traits, genetic polymorphism and evolutionary rates^32,49–52^.

## Material and Methods

### Taxon sampling and transcriptome sequencing

Nine tick species from the *Ixodes* genus (*I*. *acuminatus* Neumann 1901, *I*. *arboricola* Schulze & Schlottke 1930, *I*. *canisuga* Johnston 1849, *I*. *frontalis* Panzer 1798, *I*. *holocyclus* Neumann 1899, *I*. *hexagonus* Leach 1815, *I*. *uriae* White 1852, *I*. *ventalloi* Gil Collado 1936, and *I*. *vespertilionis* Koch 1844) were collected in the field, either directly on their animal hosts or in the habitat of the host (Table 1). These nine species plus three other *Ixodes* species for which we analysed published data sets (see below) represent six described sub-genera plus one ungrouped species (*I*. *ventalloi*). Morphological identification was conducted using keys of Hillyard^53^, Perez-Eid^54^, Barker & Walker^55^ and of ornithophilic ticks^56^. Our aim was to obtain a catalogue of transcripts as broad as possible for each species. For that purpose, whenever it was possible, we tried to collect a diversity of morphs and conditions (nymphs, females, males, and fed or unfed ticks). A laboratory stock of *I*. *arboricola* that originate from woodlands around Antwerp (Belgium) was established in 2008, and maintained during the following 8 years by allowing ticks to feed on great tits (*Parus major*)^57^. *I*. *acuminatus* was sampled in the field in 2013; its RNA was extracted along with that of *I*. *ricinus* samples as described in our recent paper^58^. All remaining species were sampled in 2016. Two ticks from more distant sampling locations (*I*. *arboricola*, *I*. *holocyclus*) were sent (dead) to our laboratory in RNAlater, in cold conditions (4 °C), whereas the other species were kept alive at 4 °C until RNA extraction. For seven of the nine *Ixodes* species (all species except *I*. *uriae* and *I*. *acuminatus*), whole tick bodies were ground in Trizol (Invitrogen, Life Technologies, Carlsbad, CA, USA). RNA was then purified keeping the aqueous phase of a centrifugation with chloroform. RNA was extracted using NucleoSpin RNA XS column (Macherey-Nagel, Düren, Germany) including a DNase and was stored in RNAsin (Promega, Madison, USA). RNA concentration and quality of each sample were determined using Nanodrop (Thermo Fisher Scientific, Waltham, USA), Qubit (Invitrogen, CA) and Agilent Bioanalyzer. For species with multiple conditions (different life stages and feeding statuses), RNA extraction was performed independently for each condition; these different samples were used to produce an equi-molar mix and a single final sample per species. After mRNA isolation using polyA Magnetic Isolation Module, cDNA libraries were prepared using the TruSeq stranded mRNA preparation kit and cDNA was sequenced on one lane of an Illumina HiSeq. 2500 machine, producing strand oriented read pairs (2 × 125bp).

For *I*. *uriae*, ticks were sampled on Hornoeya, an island in northern Norway from a colony of common murres (*Uria aalge*). Ticks were brought back to France live and maintained at 4 °C. Then they were frozen at −80 °C for 24 H, and extraction was performed using the RNeasy mini Kit of Qiagen. Adults were grouped by pools of five and nymphs by pools of ten individuals (2 pools of unfed nymphs, one pool of partially engorged nymphs). A DNase treatment was applied (Invitrogen TURBO^TM^ DNase). Quality was assessed with the same methods used the eight other species. In contrast to these other species, the different physiological states of *I*. *uriae* (unfed nymphs, unfed adult females, males, partially fed nymphs) were not mixed prior to library construction; a different library was generated for each physiological state. After mRNA isolation using a polyA Magnetic Isolation Module, libraries were prepared using the TruSeq stranded mRNA preparation kit, and sequencing was performed on one lane of a Hiseq3000 at the Get-Plage platform (Toulouse) producing strand oriented read pairs (2 × 150 bp).

We also included previously published sequences for three additional *Ixodes* species (*I*. *ricinus*, *I*. *persulcatus* and *I*. *scapularis*) as well as 15 non-*Ixodes* species of ticks in our analysis (Supplementary Table S1): while sequences corresponding to different technologies have been published, we chose to analyze only RNA-Seq projects performed with the Illumina technology for homogeneity purposes. This type of data was also the most common and typically yield a high output and high sequence accuracy. The non-*Ixodes* species were chosen to reflect the diversity of Metastriata genera (12 species of five genera included) and the outgroup (three species of the *Ornithodoros* genus). For these species, raw reads were downloaded from the Sequence Read Archive section of NCBI (Supplementary Table S1). The next steps (quality checks and *de novo* assembly) were applied to 26 of the species studied here, whereas for *I*. *ricinus*, we used an assembly previously obtained by our group with the same methods^58^.

### Reads quality check

Newly obtained sequences as well as reads obtained from NCBI (SRA division) were cleaned using the same automated approach. For all species, a first round of cleaning was performed using Trimmomatic−0.36^59^: this step removed adapters and trimmed sequences by quality, with a minimum sequence length of 36 bp. Cleaned reads were checked for quality with FastQC. If necessary, a second round of cleaning by Trimmomatic was performed until no sign of adapters could be detected from the FastQC report.

### *De novo* transcriptome assembly

Resulting reads for each of the 27 species were *de novo* assembled with Trinity (v2.2.0)^60^. For data sets with strand-oriented sequencing, the corresponding option was applied in the assembly process. Coding sequences were retrieved using Transdecoder.longOrfs (3.0.1)^60^. To suppress redundancy resulting from alternative splicing, we proceeded in two steps. First, coding sequences were clusterized by CD-HIT (v4.6) with a 95% of identity threshold at the amino acid level^61^ and only the corresponding contigs were kept. Then we extracted the longest contig by component of the Transcriptome-DeBruijn Graph (T-DBG) which further eliminated potential alternative transcripts. Several statistics were computed at each step of the pipeline to monitor the quality of our assembly and predicted gene sets. We measured completeness using BUSCO (v2 with Arthropod database v1, n = 2675 conserved elements)^62^ as well as general assembly statistics (using homemade R scripts). These novel scripts are available at http://www.github.com/npchar/Transcriptome-Assembly-Statistics/.

### Orthology prediction and gene matrix construction

Orthologous genes are defined as sequences from different species that are homologous and have diverged after speciation events. This excludes genes produced by duplication events which could alter inference of the species tree. We used SiLiX 1.2.9^63^ to infer homology relationships from an all-versus-all blastp+ (v 2.3.0)^64^ using 50 threads from the BIRD Computational Resources Center (Nantes, France). We defined orthologues as genes present in a cluster only once in each species (single copy orthologues, or SCO). The SiLiX approach needs four user-defined parameters to consider a blast hit as significant to define homology and produce a gene cluster (minimum identity, overlap percentage, minimum length and partial overlap). To find the optimal combination of SiLiX parameters (i.e. parameters maximizing the number of SCO genes present in all species), we conducted 1050 different clusterings by varying the different parameters. Based on the presence/absence of a gene in the 27 species, we defined 3 SCO matrixes with different gene occupancy levels (100%, 75%, 50%)^19^.

### SCO alignments, saturation assessment and concatenation

For each matrix (differing by gene occupancy level), SCO genes were first aligned at the protein level using clustalW (v 2.1)^65^. For DNA-level analyses, protein sequences alignments were reported on DNA sequences using a homemade perl script. Alignments were trimmed using Gblocks v091b^66^ preserving the codon structure. In order to assess the quality of DNA alignments, we investigated potential saturation produced by hidden multiple substitutions. For every SCO alignment, we calculated the pairwise p-distance and a distance corrected by the TN93 model. We then tested if the relationship between p-distance and the corrected distance tended to plateau or not^67^. Sequences that showed a deviation from a linear relationship between the two distances were discarded. For ML and Bayesian approaches, the resulting alignments were then concatenated to produce the three super-alignments (SCO present at least in 100%, 75%, or 50% of the 27 species). Finally, for the Quartet method (based on the reconstruction of supertrees), we conducted analyses for two other data sets: (i) DNA alignments containing only the first and second positions (considering that the third position is the most sensitive to saturation effects), and (ii) amino acid alignments. The alignments used for this analysis were the nucleotide alignments produced as described above, which were either translated or in which third positions were deleted.

### Nuclear genes-based phylogeny

Species relationships were inferred with three different approaches. Maximum Likelihood inferences were made on the three concatenated supermatrices with IQ-TREE^68^ with the best estimated partition sites^69^ and under the best model of substitution per partition^70^ as well as 1000 ultrafast bootstrap estimation^71^. Bayesian Inferences were made using a CAT-GTR model implemented in Phylobayes^72^ removing constant sites. The convergence of the Bayesian sampler was checked by comparing two independent runs for every supermatrix. We used two programs associated with Phylobayes, tracecomp and pbcomp^73^ to assess the convergence of the 8 parameters of the trace and the bipartition list. The usual minimum criteria for satisfactory convergence are an effective size greater than 50 and a maximum discrepancy of 0.3. Super-networks were constructed using a mixture of gene trees, based on a quartet approach as implemented in the SuperQ software^74^. For the super-network approach, SCO gene trees were independently inferred with IQ-TREE^68^ under the best model of substitution^70^ with 100 classic non-parametric bootstraps. The best model among those tested was chosen according to the Bayesian Information Criterion (BIC). The resulting trees were divided in quartets (subtrees of 4 taxa) and assembled together with SuperQ^74^ to produce a planar supernetwork with the Gurobi Optimizer. An advantage of this method is to scale input trees in order to keep information of branch lengths. Supernetworks were reconstructed for the three different gene occupancy levels (100%, 75%, and 50%) for three data sets: (i) nucleotide level alignments including all codon positions, (ii) nucleotide level alignments including only the first and second codon positions, and (iii) amino-acid level alignments. Finally, genetic distance among among genuses were calculated: we used the patristic distance, defined as the sum of branch lengths between taxa and calculated with the R function distTips.

### Mitochondrial genes-based phylogeny

A specific analysis was also performed on mitochondrial (mt) genes, in order to build a mtDNA-based phylogeny and use it as an additional phylogenetic data set. For each species included in our study, we checked for the presence of a published mitogenome and used it if available. For species with several mitogenomes, corresponding to different lineages, we used the accessions with highest identity to mt genes reconstructed from our transcriptome assemblies (for example the sequence KF197132 for *I*. *ricinus*, and KJ133594 for *O*. *moubata*). A list of the sequences used is given in Supplementary Table S2: this list includes four species not studied in the nuclear genes-based phylogeny (because no transcriptome was available for them), *I*. *rubicundus*, *I*. *pavlovskyi*, *I*. *simplex*, *I*. *tasmani*. When no published mitogenome was available, mitochondrial gene sequences were extracted from RNA-Seq contigs. Given the difference in genetic code (invertebrate mitochondrial versus standard code), we could not use the collections of predicted CDSs to extract mt genes. Rather, we searched contigs by a tblastn of each mitochondrial protein (using different published tick mitogenomes as queries), and selecting the corresponding genetic code (i.e. “invertebrate mitochondrial”). We did this search for the nine longest mt gene sequences; in fact, we also tried to extract gene sequences for the next longest sequence (*nad6*) but had to discard it because it was too partial in several of the assembled transcriptomes. We also decided to remove the three shortest sequences (*nad3*, *nad4L*, *atp8*) as they contained too little information. We often found several contigs patching each gene in each assembly (i.e. species). For example we usually found two contigs in opposite strand but with an identical ORF. We also found additional divergent sequences, including for example mt sequences from the vertebrate host (a bird or a mammal species), or even other contaminants (e.g. human DNA). Finally a few of the partial sequences reflected patterns of cross-contaminations among libraries. For the libraries generated by our project, we indeed detected this phenomenon after finding contigs corresponding to the mt gene sequence of an *Ixodes* species that differed from the focal library/sample. A likely explanation is index hopping, a phenomenon which consists in miss-assignment of reads between multiplexed libraries (appearing as an apparent cross-species contamination among assembled transcriptomes). We evaluated this phenomenon quantitatively by counting reads mapping to sequences of the different species in each library: we found that the level of cross-mapping was always less than 0.1%, the corresponding assembled sequences being usually only partial. We thus performed a phylogenetic analysis at the amino acid level for nine mt sequences (*atp6*, *cox1*, *cox2*, *cox3*, *cytb*, *nad1*, *nad2*, *nad4*, *nad5*), amounting to 89% of the cumulated length of mitochondrial protein coding genes. For each of these genes, we obtained complete, or nearly complete sequences in all species (see Results). For each gene, ORF sequences were translated and aligned using ClustalW; the nine mt gene sequences were concatenated (forming a data set hereafter referred to as mtAA9, which comprises 3,188 amino acid positions). We then performed an analysis with IQ-TREE^68^ to: (i) determine an optimal model of substitution with Model Finder^70^ as described above for nuclear genes, (ii) infer a ML-phylogeny using the edge-linked partition model in IQ-TREE, and the optimal model inferred for each of the nine protein sequences^69^ and (iii) assess branch support with 1000 ultrafast bootstrap replications^71^.

### Comparing rates of substitution

To test for a possible difference in evolutionary rates among species, we used Tajima’s relative rate test^75^. This test uses counts of substitutions unique to each of two focus species (A and B), using a third species as an as an outgroup. These comparisons were performed after observing particular long branches in the phylogenetic analyses, and concerned (i) a comparison between Prostriata and Metastriata ticks, using a soft tick species as an outgroup (ii) a comparison between species of the *Rhipicephalus* genus -comparing the Boophilus subgenus to other species - using another species of Metastriata as the outgroup.

## Supplementary information

Supplementary Information

## Data Availability

Reads from newly sequenced transcriptomes were deposited into the SRA section of the NCBI under BioProject accessions PRJNA528282 and PRJNA550328. DNA alignments of the SCO50 matrix (nuclear genes) are available at 10.5281/zenodo.3382009.
