# Supplementary material for: A transcriptome-based phylogenetic study of hard ticks (Ixodidae)
Source: Sci Rep. 2019 Sep 9;9:12923. doi: 10.1038/s41598-019-49641-9 (PMC6733903; doi:10.1038/s41598-019-49641-9)
Supplement: Supplementary file 1 — Supplementary Information [file 41598_2019_49641_MOESM1_ESM.pdf]

Supplementary Information for

A transcriptome-based phylogenetic study of hard ticks (Ixodidae).

N. Pierre Charrier, Axelle Hermouet, Caroline Hervet, Albert Agoulon, Stephen C. Barker, Dieter Heylen, Céline Toty, Karen D. McCoy, Olivier Plantard, Claude Rispe

Supplementary Figures

Supplementary Figure S1: Completeness metrics for the 27 transcriptome assemblies. Species are represented in row, different colors representing the result of a BUSCO search (Arthropod BUSCO v1 data set, n=2675 genes), with four categories: i) genes complete and single copy (Complete), ii) genes complete and multi-copy (Complete and Duplicated), iii) genes with an incomplete sequence (Fragmented), and iv) genes not found (Missing).

Supplementary Figure S2: Completeness as a function of depth of sequencing. Y-axis, number of complete BUSCO genes (Complete or Duplicated), x-axis, number of pair of reads used for *de-novo* assembly.

Supplementary Figure S3: Number of SCO detected by SiLiX for different overlap and identity parameters. Each line represents the number of SCO detected at three thresholds of occupancy (respectively for 100%, 75% and 50%). The first column represents the number of SCO as a function of the percentage of overlap for an identity fixed at 75%. The second column represents the number of SCO as a function of identity and overlap. The third column represents the number of SCO as a function of the percentage of identity for an overlap fixed at 75%. A red vertical line represents the chosen parameters for the final SCO detection (identity of 75%, overlap of 75%).

Supplementary Figure S4: Maximum Likelihood molecular phylogenetic analysis for the mitochondrial *cox1* gene (1536 positions), for a subset of *Ixodes* species. This analysis includes two of the most divergent lines of *I. ricinus* in the study of Carpi and coauthors (Carpi et al., 2016), and two divergent variants of *I. frontalis* . For *I. frontalis*, the two sequences were reconstructed after a careful and stringent reassembly of the reads of our newly sequenced transcriptome (initial contigs obtained with Trinity were chimeric). The model was Tamura Nei (+G, parameter = 0.4996 and +I, 52.1689% sites). Values at the nodes indicate bootstrap support. Evolutionary analyses were conducted with MEGA7 (Kumar et al., 2016).

Supplementary Figure S5: Maximum Likelihood molecular phylogenetic analyses for two regions of the mitochondrial *cytb* gene.The alignment included the first mitogenome to be published for *I. ricinus* (JN248424), which was found to be very divergent from other mitogenomes of this species, in particular in the *cytb* sequence (Montagna et al., 2012). The alignment and ML phylogeny were conducted separately for two regions, due to an apparent shift in divergences before and after position 490. Above: phylogeny corresponding to the position 1-489 of the alignment. Below, phylogeny corresponding to the position 490-1080 of the alignment.

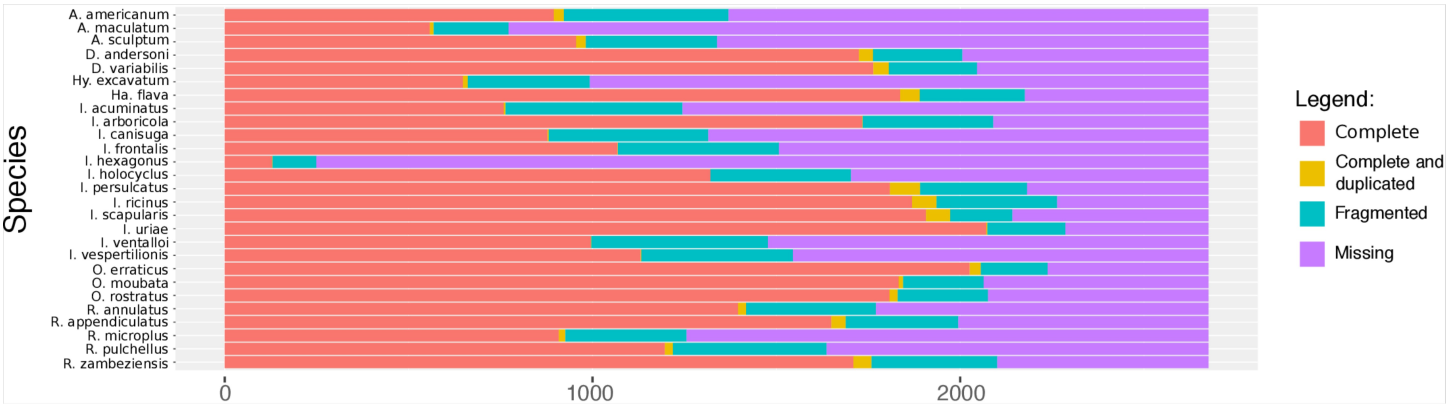

Supplementary Figure S1

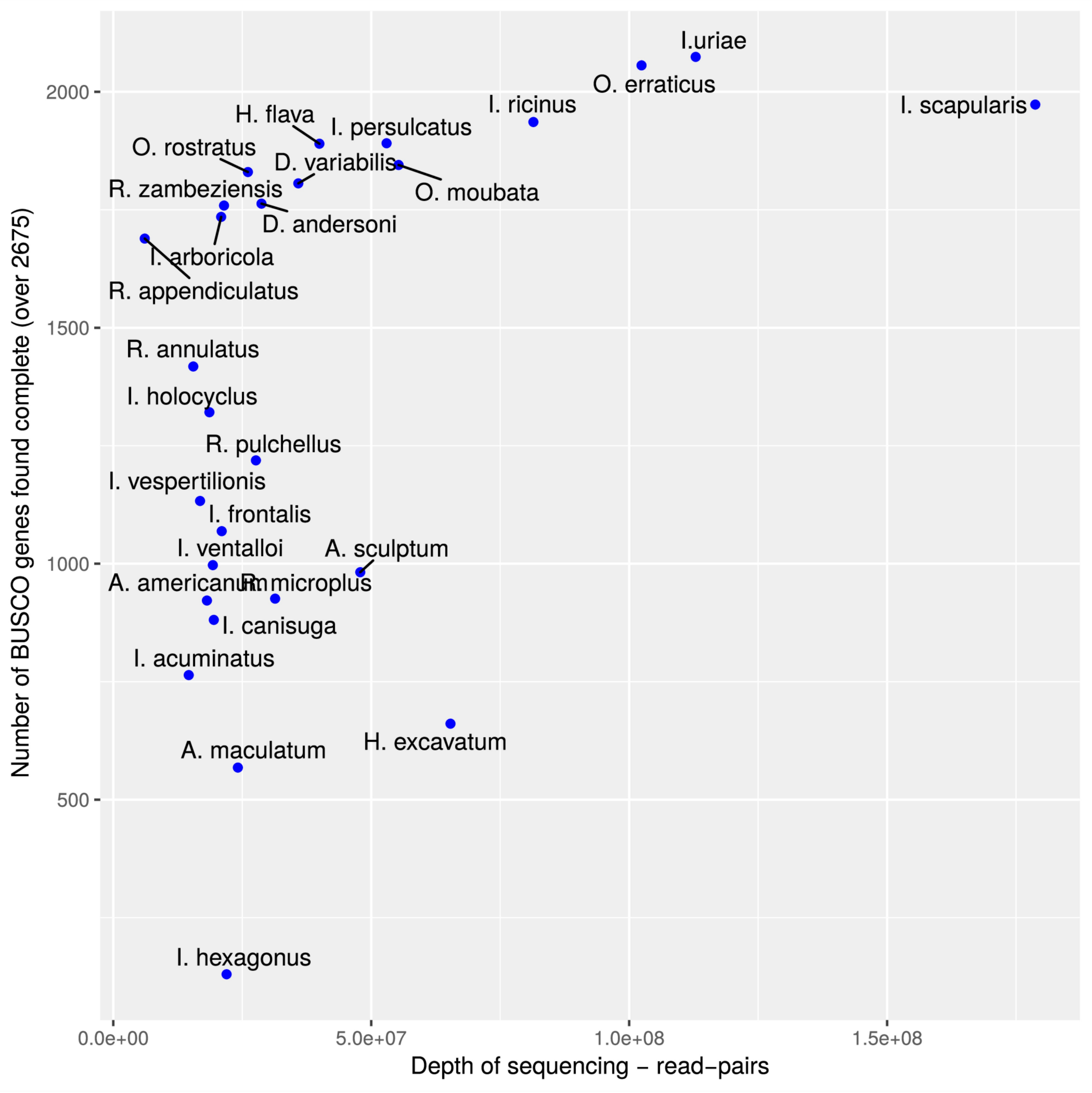

Supplementary Figure S2

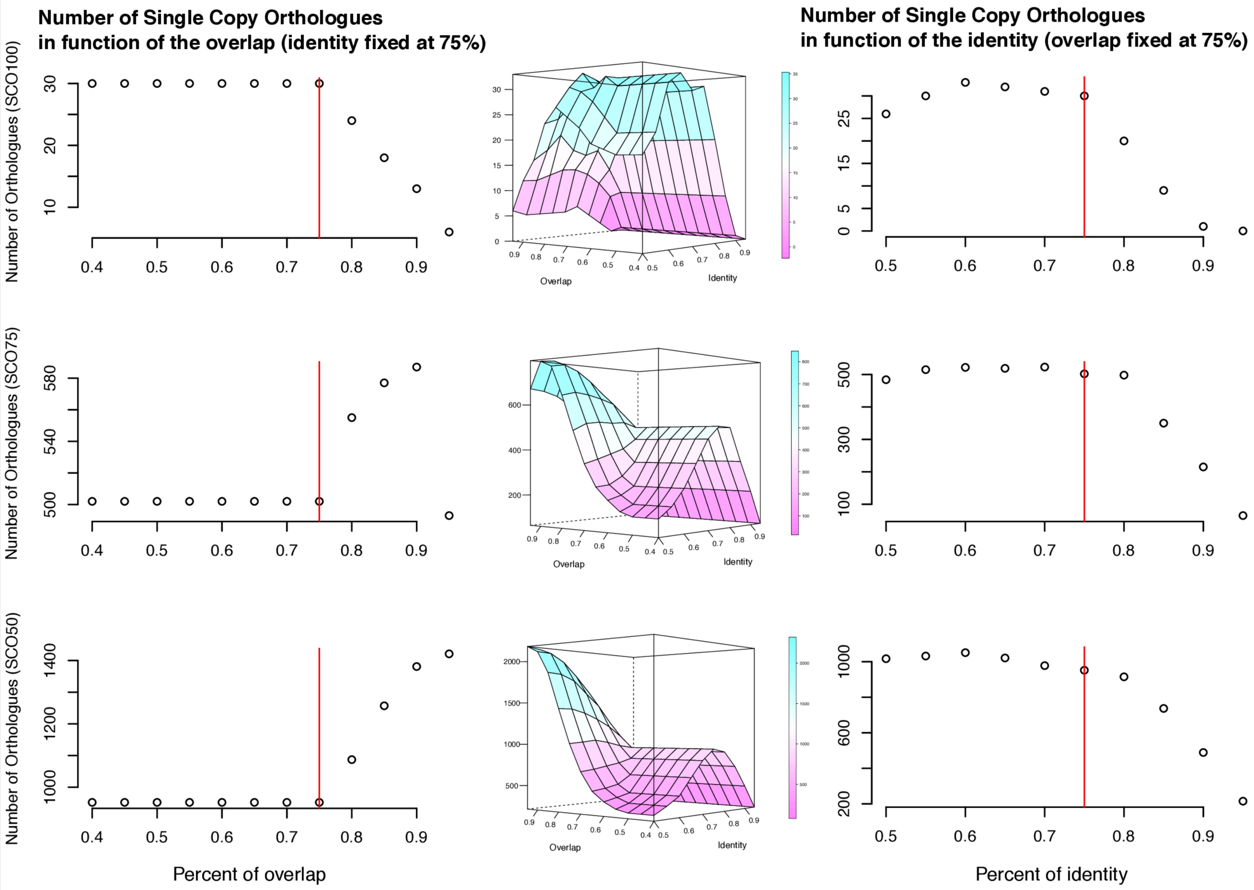

Supplementary Figure S3

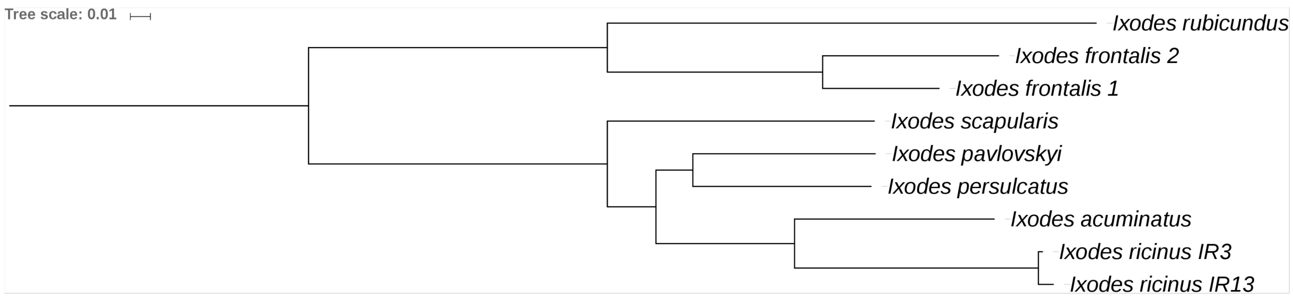

Supplementary Figure S4

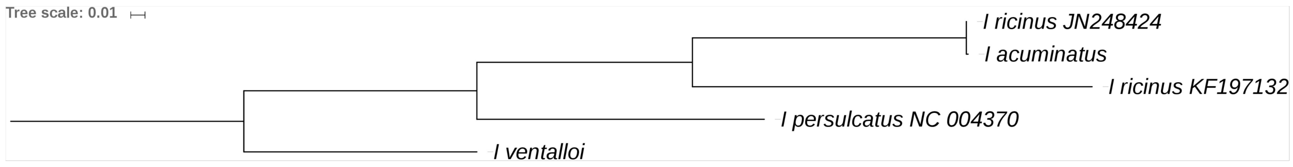

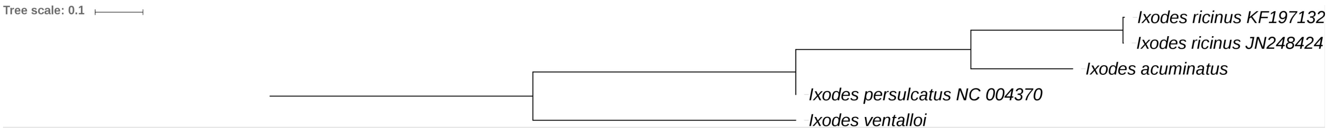

Supplementary Figure S5

Supplementary Table S1: List of species for which public high-throughput RNA-Seq datasets were used to produce a transcriptome-based phylogeny. From left to right, species name, SRA accession numbers (accessions separated by a trait mean that all consecutive numbers were used), sequencing platform, read length (before trimming), and total number of cleaned reads used for *de novo* assembly.

Species	SRA accession	Platform	Read length	Number of reads
A. americanum	SRR1756275	HiSeq 2000	76	11674238
A. americanum	SRR1027479, SRR1027481, SRR1027483, SRR1027751, SRR1027752, SRR1027761-SRR1027770	HiSeq 2000	100	24623364
A. maculatum	SRR959015	HiSeq 2000	101	51869140
A. sculptum	SRR3120106, SRR3126275	HiSeq 2000	90	98553100
D. andersoni	SRR1756052, SRR1756083,SRR1756090, SRR1756115, SRR1764995, SRR1764997, SRR1765017, SRR1765088, SRR1765089, SRR1765224, SRR1765260	HiSeq 2000	76	69688944
D. variabilis	SRR1755788, SRR1755810, SRR1755886, SRR1756051, SRR1756250, SRR1756258, SRR1756312, SRR1756365, SRR1756419, SRR1756426, SRR1756485, SRR1765096, SRR1765148, SRR1765247	HiSeq 2000	76	83241616
H. excavatum	SRR3157671, SRR3157672	HiSeq 2000	101	138144530
H. flava	SRR2057872, SRR2057873	HiSeq 2500	125	120473540
O. erraticus	SRR6012550-SRR6012553	Genome Analyzer II	125	235010350
O. moubata	SRR5313934	Genome Analyzer II	125	121695122
O. rostratus	SRR1732011, SRR1732013	HiSeq 2000	101	53889866
R. annulatus	SRR1522091, SRR1522109, SRR1522120	Genome Analyzer IIx	116	62279268
R. appendiculatus	SRR2568018	MiSeq	246	26432764
R. microplus	SRR1187010	HiSeq 1000	100	72356116
R. pulchellus	SRR521835	HiSeq 2000	101	72415764
R. zambeziensis	SRR5438376, SRR521951	MiSeq	300	45306680
I. persulcatus	SRR1598812,SRR1598845, SRR1598940	HiSeq 2000	102	59181716
I. persulcatus	SRR1604864	Genome Analyzer II	91	53579464
I. ricinus	SRR5839696-SRR5839710	HiSeq 2500	125	162872698
I. scapularis	SRR1189650-SRR1189652	HiSeq 2500	102	410147172

Supplementary Table S2: List of accessions for published mitogenome sequences included in our mitochondrial genes-based phylogenetic analysis. We used the same 27 species included in the transcripome-based phylogenetic reconstruction and four more *Ixodes* species marked by an asterisk: *I. pavlovskyi*, *I. rubicundus*, *I. simplex*, *I. tasmani* (Burnard and Shao, 2019). For species without a published mitogenome sequence (noted "na"), mitochondrial gene sequences were extracted from the RNA-Seq contigs as described in the text.

Species	Mitogenome accession
Amblyomma americanum	NC_027609
Amblyomma maculatum	na
Amblyomma sculptum	NC_032369
Dermacentor andersoni	na
Dermacentor variabilis	na
Haemaphysalis flava	NC_005292
Hyalomma excavatum	na
Ixodes acuminatus	na
Ixodes arboricola	na
Ixodes canisuga	na
Ixodes frontalis	na
Ixodes hexagonus	NC_002010
Ixodes holocyclus	NC_005293
*Ixodes pavlovskyi**	NC_023831
Ixodes persulcatus	NC_004370
Ixodes ricinus	KF197132
*Ixodes rubicundus**	KY457530
Ixodes scapularis	na
*Ixodes simplex**	KY457531
*Ixodes tasmani**	MH043271
Ixodes uriae	NC_006078
Ixodes ventalloi	na
Ixodes vespertilionis	na
Ornithodoros erraticus	na
Ornithodoros moubata	KJ133594
Ornithodoros rostratus	KC769592
Rhipicephalus annulatus	na
Rhipicephalus appendiculatus	KY457535
Rhipicephalus microplus	KP143546
Rhipicephalus pulchellus	na
Rhipicephalus zambeziensis	KY457543

Supplementary Table S3: Detailed statistics of the Baysian phylogenetic analysis, for nuclear genes. Effective sizes (between parentheses, maximum discrepancies between the two chains) for a set of eight parameters (lines) and for the three supermatrices (SCO100, SCO75, SCO50). Chain 1 and Chain 2 are the numbers of iterations achieved. MaxDiff and MeanDiff indicate the convergence of the bipartition list.

	SC0100	SCO75	SCO50
loglik	4744 (0.0127)	2114 (0.0292)	206 (0.1819)
length	4846 (0.0279)	15063 (0.0129)	2046 (0.0213)
alpha	9095 (0.0097)	19842 (0.0003)	2129 (0.0010)
Nmode	1687 (0.0134)	503 (0.0591)	400 (0.2097)
statent	4486 (0.0133)	3004 (0.0204)	233 (0.1396)
statalpha	6595 (0.0332)	3672 (0.0030)	689 (0.1590)
rrent	10987 (0.0072)	20279 (0.0019)	19443 (0.0173)
rrmean	14556 (0.0144)	1578 (0.0251)	11138 (0.0133)
Chain 1	22275	42835	21769
Chain 2	22306	40167	21442
MaxDiff	0.0315	0.0202	0.0067
MeanDiff	0.0010	0.0003	0.0001

Supplementary Table S4: Compared evolutionary rates between different groups, for mitochondrial or nuclear genes. This table reports statistics of the Tajima's relative rate test for species of different subgroups (respectively A and B), a third species being used as an outgroup - tests performed using MEGA7 (Kumar et al., 2016). Columns: substitutions identicall in all species, divergent in all species, unique in a subgroup, or unique in the outgroup, significance, ratio between the numbers of substitutions unique in A versus substitutions unique in B.

Species A	Species B	outgroup (C)	Identical in all	Divergent in all	Unique in A	Unique in B	Unique in C	Significance	Ratio A/B
a) Mitochondrial genes (n=9), Metastriata vs Prostriata									
I. canisuga	*R. microplus*	*O. rostratus*	1569	588	238	394	362	P=0.00000	1.66
I. canisuga	*A. americanum*	*O. rostratus*	1594	572	228	369	387	P=0.00000	1.62
I. canisuga	*D. variabilis*	*O. rostratus*	1566	602	227	398	357	P=0.00000	1.75
I. canisuga	*Hae. flava*	*O. rostratus*	1597	555	216	366	416	P=0.00000	1.69
I. ricinus	*R. microplus*	*O. rostratus*	1561	584	247	390	372	P=0.00000	1.58
I. ricinus	*A. americanum*	*O. rostratus*	1584	564	239	367	399	P=0.00000	1.54
I. ricinus	*D. variabilis*	*O. rostratus*	1565	596	228	384	376	P=0.00000	1.68
I. ricinus	*Hae. flava*	*O. rostratus*	1589	561	225	361	417	P=0.00000	1.60
I. frontalis	*R. microplus*	*O. rostratus*	1570	626	238	365	358	P=0.00000	1.53
I. frontalis	*A. americanum*	*O. rostratus*	1584	597	240	352	383	P=0.00000	1.47
I. frontalis	*D. variabilis*	*O. rostratus*	1563	617	233	372	367	P=0.00000	1.60
I. frontalis	*Hae. flava*	*O. rostratus*	1596	587	221	339	413	P=0.00000	1.53
I. uriae	*R. microplus*	*O. rostratus*	1550	615	257	383	348	P=0.00000	1.49
I. uriae	*A. americanum*	*O. rostratus*	1584	609	239	350	369	P=0.00000	1.46
I. uriae	*D. variabilis*	*O. rostratus*	1557	628	236	375	352	P=0.00000	1.59
I. uriae	*Hae. flava*	*O. rostratus*	1586	597	227	348	393	P=0.00000	1.53
b) Nuclear genes (n=30), Metastriata vs Prostriata									
I. canisuga	*R. microplus*	*O. rostratus*	5554	209	216	261	400	P=0.03936	1.21
I. canisuga	*A. americanum*	*O. rostratus*	5601	200	217	214	408	P=0.88510	0.99
I. canisuga	*D. variabilis*	*O. rostratus*	5578	178	218	237	429	P=0.37307	1.09
I. canisuga	*Hae. flava*	*O. rostratus*	5607	202	204	208	419	P=0.84378	1.02
I. ricinus	*R. microplus*	*O. rostratus*	5577	194	193	265	411	P=0.00077	1.37
I. ricinus	*A. americanum*	*O. rostratus*	5618	186	200	224	412	P=0.24380	1.12
I. ricinus	*D. variabilis*	*O. rostratus*	5604	164	192	238	442	P=0.02653	1.24
I. ricinus	*Hae. flava*	*O. rostratus*	5623	181	188	219	429	P=0.12439	1.16
I. frontalis	*R. microplus*	*O. rostratus*	5568	196	202	269	405	P=0.00202	1.33
I. frontalis	*A. americanum*	*O. rostratus*	5609	187	209	228	407	P=0.36341	1.09
I. frontalis	*D. variabilis*	*O. rostratus*	5595	166	201	242	436	P=0.05142	1.20
I. frontalis	*Hae. flava*	*O. rostratus*	5612	186	199	225	418	P=0.20671	1.13
I. uriae	*R. microplus*	*O. rostratus*	5529	209	241	255	406	P=0.52960	1.06
I. uriae	*A. americanum*	*O. rostratus*	5567	192	251	217	413	P=0.11603	0.86
I. uriae	*D. variabilis*	*O. rostratus*	5557	186	239	227	431	P=0.57829	0.95
I. uriae	*Hae. flava*	*O. rostratus*	5568	197	243	216	416	P=0.20458	0.89
c) Mitochondrial genes (n=9), Boophilus vs other *Rhipicephalus* sp.									
R. pulchellus	*R. microplus*	*Hy. excavatum*	2177	243	119	183	432	P=0.00023	1.54
R. appendiculatus	*R. microplus*	*Hy. excavatum*	2159	248	137	195	416	P=0.00146	1.42
R. pulchellus	*R. annulatus*	*Hy. excavatum*	2180	248	115	179	429	P=0.00019	1.56
R. appendiculatus	*R. annulatus*	*Hy. excavatum*	2161	249	134	192	416	P=0.00132	1.43
d) Nuclear genes (n=30), Boophilus vs other *Rhipicephalus* sp.									
R. pulchellus	*R. microplus*	*Hy. excavatum*	6367	20	68	125	60	P=0.00004	1.84
R. appendiculatus	*R. microplus*	*Hy. excavatum*	6382	18	70	125	45	P=0.00008	1.79
R. pulchellus	*R. annulatus*	*Hy. excavatum*	6369	20	66	125	60	P=0.00002	1.89
R. appendiculatus	*R. annulatus*	*Hy. excavatum*	6382	17	70	124	47	P=0.00011	1.77

Supplementary Table S5: Key of the short names used in the supernetwork phylogeny (Fig.3). Short name and complete species name.

Short Name	Species name
AAMER2	*A. americanum*
AMACU	*A. maculatum*
ASCUL	*A. sculptum*
DANDE	*D. andersoni*
DVARI	*D. variabilis*
HFLAV	*Ha. flava*
HEXCA2	*Hy. excavatum*
IACUM	*I. acuminatus*
ARBO05	*I. arboricola*
CANI04	*I. canisuga*
FRON06	*I. frontalis*
HEXA01	*I. hexagonus*
HOLO07	*I. holocyclus*
IPERS	*I. persulcatus*
IRIC	*I. ricinus*
ISCAP	*I. scapularis*
IURIAE	*I. uriae*
VENT03	*I. ventalloi*
VESP02	*I. vespertilionis*
OERRA	*O. erraticus*
OMOUB	*O. moubata*
OROST	*O. rostratus*
RANNU	*R. annulatus*
RAPPE	*R. appendiculatus*
RMICR	*R. microplus*
RPULC	*R. pulchellus*
RZAMB	*R. zambeziensis*
